# Biomimetic Tactile Sensors with Bilayer Fingerprint Ridges Demonstrating Texture Recognition

**DOI:** 10.3390/mi10100642

**Published:** 2019-09-25

**Authors:** Eunsuk Choi, Onejae Sul, Jusin Lee, Hojun Seo, Sunjin Kim, Seongoh Yeom, Gunwoo Ryu, Heewon Yang, Yoonsoo Shin, Seung-Beck Lee

**Affiliations:** 1 Department of Electronic Engineering, Hanyang University, 222 Wangsimni-ro, Seongdong-gu, Seoul 04763, Korea; silver77@hanyang.ac.kr (E.C.); jusin19@hanyang.ac.kr (J.L.); masiks@hanyang.ac.kr (H.S.); akangel0307@gmail.com (S.K.); yso526@hanyang.ac.kr (S.Y.); rgwrgw00@gmail.com (G.R.); heewon0820@hanyang.ac.kr (H.Y.); skstls715@hanyang.ac.kr (Y.S.); 2 Institute of Nano Science and Technology, Hanyang University, 222 Wangsimni-ro, Seongdong-gu, Seoul 04763, Korea; ojsul@hanyang.ac.kr

**Keywords:** biomimetic, tactile sensor, fingerprint ridge, piezoelectric sensor, texture discrimination

## Abstract

In this article, we report on a biomimetic tactile sensor that has a surface kinetic interface (SKIN) that imitates human epidermal fingerprint ridges and the epidermis. The SKIN is composed of a bilayer polymer structure with different elastic moduli. We improved the tactile sensitivity of the SKIN by using a hard epidermal fingerprint ridge and a soft epidermal board. We also evaluated the effectiveness of the SKIN layer in shear transfer characteristics while varying the elasticity and geometrical factors of the epidermal fingerprint ridges and the epidermal board. The biomimetic tactile sensor with the SKIN layer showed a detection capability for surface structures under 100 μm with only 20-μm height differences. Our sensor could distinguish various textures that can be easily accessed in everyday life, demonstrating that the sensor may be used for texture recognition in future artificial and robotic fingers.

## 1. Introduction

The human ability of tactile sensing using the finger plays an essential role in object manipulation and our interaction with the external environment. When making contact with an object, we can recognize its texture using tactile senses made through mechanoreceptors distributed throughout the skin [1]. Therefore, research into tactile sensing attempts to mimic the mechanoreceptors in the human skin, their pressure sensing ability, and their distribution in the skin. Recently, many groups have reported on pressure sensors that demonstrate basic artificial tactile feeling and texture pattern recognition for prosthesis and robotics applications. They have placed pressure sensors at the digits of robotic arms to grip and manipulate objects and have arranged pressure sensors in an array form to detect the shape of an object [2,3,4,5]. Electronic skin instrumented with pressure sensors for prosthesis application has also been reported [6,7,8].

Many attempts at improving tactile sensing ability have focused on enhancing pressure sensing sensitivity in the belief that higher pressure sensitivity would lead to enhanced sensibility of the contacting object. To enhance sensitivity of the pressure sensors, a capacitor-type pressure sensor was demonstrated, which was able to detect only a few milligrams of tactile pressures [9,10,11,12,13,14,15]. However, the high pressure sensitivity was achievable only at low spatial resolution, limiting the fine-texture detecting capability. A closer inspection of the human skin showed that the sensitivity and distribution of the mechanoreceptors are not the only criteria for tactile sensing.

When a human finger slides over an object’s surface, the fingerprint ridge structures interact with the object’s surface structure and generate vibrational signals, which are then transmitted to the mechanoreceptors lying under the epidermis. The generated action potential pulses in the mechanoreceptors travel to the brain, where they are interpreted as shear interaction and are used for recognizing the “fine texture” of the object [16]. To detect fine surface textures, ridge structures mimicking human fingerprint ridges have been applied to tactile sensors [17,18,19,20,21]. The biomimetic ridge structures showed the ability to detect a few hundred micrometers of scale surface structures [18], and it was found that biomimetic ridge structures are an essential element for material and textural identification. Despite there being many studies on tactile sensors with biomimetic ridge structures, only a few have reported studies on the ridge structure itself, e.g., elasticity, dimension, for the improvement of texture recognition.

In this study, we developed a biomimetic tactile sensor with a surface kinetic interface (SKIN) that imitates two elements of the human skin: epidermal fingerprint ridges and the epidermis. Our tactile sensor is composed of a SKIN (biomimetic epidermal fingerprint ridges and supporting epidermal board) layer and a polyvinylidene difluoride (PVDF) piezoelectric polymer layer that acts as a vibration sensor, which functions similarly to a fast-adapting mechanoreceptor. We evaluated the effectiveness of the SKIN layer in transferring surface shear forces into vertical vibrations while varying the elasticity and geometrical factors of the epidermal fingerprint ridges and epidermal board. We found that the tactile sensitivity was increased when using a softer epidermal board and a harder epidermal fingerprint ridge. The biomimetic tactile sensor with SKIN showed detectability for sub-100-μm surface structures and 20-μm height differences. Our sensor produced signals that made it possible to discern different types of paper, leather, and fabrics. It was also possible to distinguish a real human finger from its replica. The developed biomimetic tactile sensor may be applied to future artificial fingers for texture detection and recognition.

## 2. Surface Kinetic Interface (SKIN)

**Figure 1:**
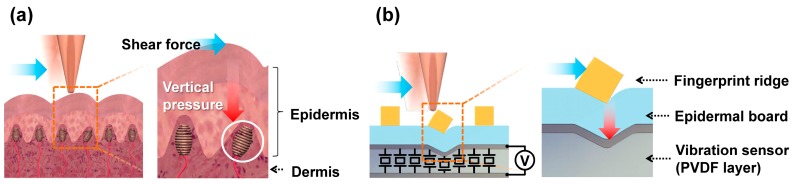
Schematic illustration and shear detecting mechanism of (**a**) the human fingertip and (**b**) the biomimetic tactile sensor.

An illustration of human skin on a fingertip can be seen in Figure 1a. From a tactile sensing perspective, the human skin is composed of two parts, the epidermis and the dermis. The epidermis comes in contact with the outside world and transfers kinetic interactions to the dermis. In the dermis, various mechanoreceptors convert the mechanical stimulus into action potential signals. The mechanoreceptor illustrated in Figure 1a is a Meissner’s corpuscle, which generates an action potential responding to changes in the surface strain [22]. In the finger epidermis, the top layer forms epidermal fingerprint ridges (ERs) that interact directly with the contact material. Underneath it, there are layers of cells that we call the epidermal board that collectively act as a force transfer layer [23]. We designed our tactile sensor skin to functionally mimic the outer skin of the human fingertip: the SKIN layer that consists of ERs on top of a supporting epidermal board. The PVDF piezoelectric thin-film layer acts as an array of vibration sensors mimicking fast-adapting mechanoreceptors (see Figure 1b). The way shear forces are detected in our tactile sensor is quite similar to the human fingertip. When an object horizontally touches the SKIN surface, a lateral shear force is applied to the ERs. Torque is then generated at the contact point between the ERs, with the epidermal board acting as an axis of rotation. Therefore, the contact shear force is transformed into torque and then transferred as vertical pressure to the PVDF layer. The PVDF layer is a piezoelectric material that generates a voltage signal when applied pressure deforms its polarized molecular structures. The magnitude of the generated voltage signal depends on the magnitude of the lateral shear force; as higher shear force induces higher vertical pressure on the PVDF. In addition, the frequency of the voltage signal depends on the frequency of contact with the ERs. This depends on the relative speed of the scanning object and the period of surface protrusions on the object’s surface that makes contact with the ERs. Therefore, the magnitude and frequency of the generated output voltage depend mostly on the surface roughness and hardness. This will be further elaborated in Section 4. Although our tactile sensor is biomimetic in the SKIN structure, there are differences from human skin in that unlike each mechanoreceptor individually detecting the input stimulus, the PVDF layer acts as a continuous distribution of sensors, with the detected output signal being a parallel integration of all the voltage signals generated over the whole surface of the sensor at that particular time interval. In mapping the surface topography of a contacting material, this would be disadvantageous, since any information generated through the spatial distribution of contact pressure points within the sensor area would not be distinguishable. However, it will be shown that for detecting the texture of the contacting surface, the parallel integration of the voltage signals becomes advantages. This sensing mechanism is similar to vibrotactile sensation in the duplex theory of human tactile perception using fast-adapting mechanoreceptors [24].

## 3. Fabrication

**Figure 2:**
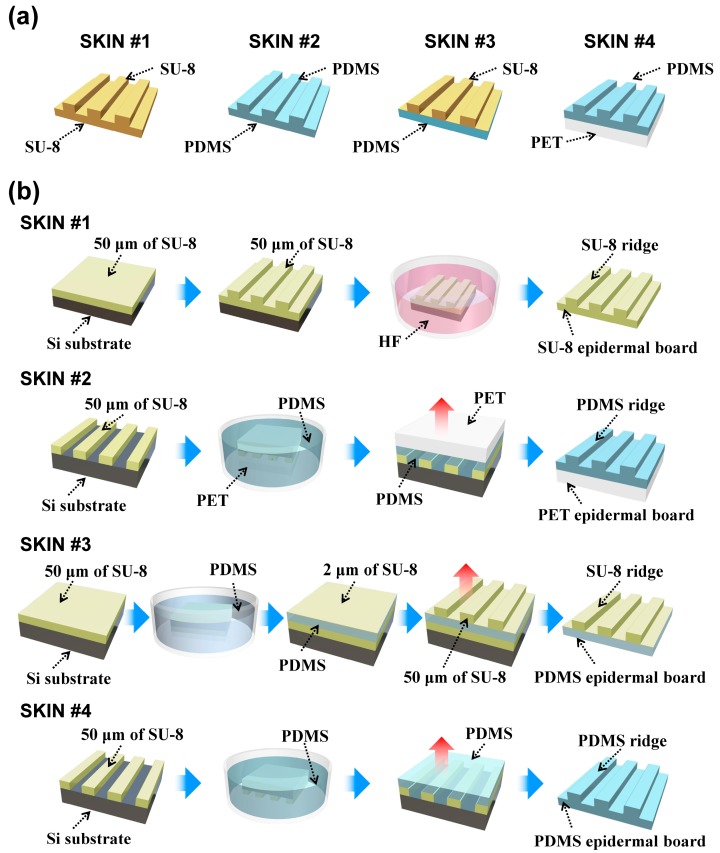
(**a**) Four types of surface kinetic interfaces (SKINs) with differing mechanical configurations and (**b**) their fabrication processes.

The Young’s modulus of human fingerprint ridges is known to be about three times higher than that of the epidermal board, which is believed to increase the durability of the outer skin layer [25]. To mimic this configuration of the human epidermis, we fabricated harder ERs using SU-8 (Microchem, Westborough, MA, USA), which had a Young’s modulus of ~3 GPa, and a soft epidermal board (using polydimethylsiloxane (PDMS)), which had a Young’s modulus of ~750 kPa. For efficient force transfer, ERs should not absorb the contact force through elastic deformation, while the epidermal board should be flexible enough to localize the deformation and allow the ER to tilt with the applied contact shear force. To test the effectiveness of this configuration, we fabricated four types of SKINs with differing mechanical configurations: hard or soft ERs and hard or soft epidermal boards. We used SU-8 and polyethylene terephthalate (PET) as the harder material (*E* ≈ 3 GPa) and PDMS as the softer material (*E* ≈ 750 kPa) (see Figure 2a). The geometry of all SKINs was identical, with the thickness of the epidermal board at 50 μm, the height of the ER at 50 μm, and the width of the ER plateau and valley both at 100 μm, giving the fingerprint pattern a 200-μm period, which is roughly half of the period of human fingerprints (0.3–0.5 mm) [26].

Figure 2b shows the fabrication processes of the different SKINs. For SKIN #1, an epidermal board was fabricated by spin-coating 50-μm-thick SU-8 on an oxidized silicon substrate. Then the SU-8 2075 was coated again at a 50-μm thickness, and ERs were patterned by using optical lithography. Then the fabricated SKIN #1 was lifted off through HF etching of the oxide underneath. For SKIN #2, a 50-μm-thick SU-8 ER master was patterned using optical lithography. A PET film was attached to the ER master and was dipped into the PDMS. The viscosity of the PDMS was low enough to fill the vacant spaces between the SU-8 master and the PET film. After the PDMS was cured, the fabricated SKIN #2 was peeled off from the master. The PET film for SKIN #2 was etched by CF_4_ physical plasma to form nanobrush structures, which promoted adhesion with PDMS by increasing the contact surface area [27,28]. For SKIN #3, a 50-μm-thick SU-8 layer and 50-μm-thick PDMS were coated on the oxidized silicon substrate. This SU-8 layer was helpful for the separation of the PDMS layer from the substrate after completion of SKIN #3. Then the surface of the PDMS was treated by oxygen plasma for better adhesion with a 2-μm-thick SU-8 layer coated on PDMS. This SU-8 thin layer works as the adhesive layer between the PDMS and the following SU-8 ERs. A 50-μm-thick SU-8 ER was patterned by using optical lithography. Finally, the fabricated SKIN #3 was peeled off from the bottom SU-8 layer on the substrate. For SKIN #4, a 50-μm-thick SU-8 ER master was patterned, and 50-μm-thick PDMS was coated on the ER master. After the PDMS was cured, SKIN #4 was peeled off.

**Figure 3:**
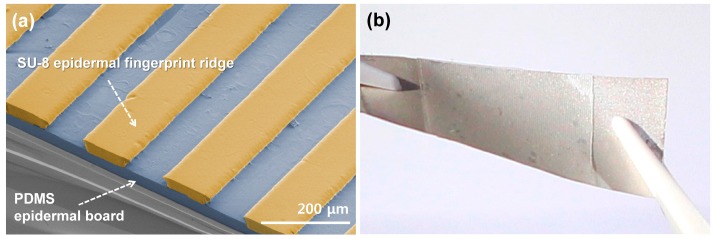
Images of a fabricated biomimetic tactile sensor with SKIN: (**a**) a false-color SEM image of the fabricated SKIN #3, which was composed of (yellow) SU-8 epidermal fingerprint ridges with a 200-μm-period and a 50-μm height, and a (blue) polydimethylsiloxane (PDMS) epidermal board with a 50-μm thickness; (**b**) an optical image of the fabricated flexible biomimetic tactile sensor with SKIN.

**Figure 4:**
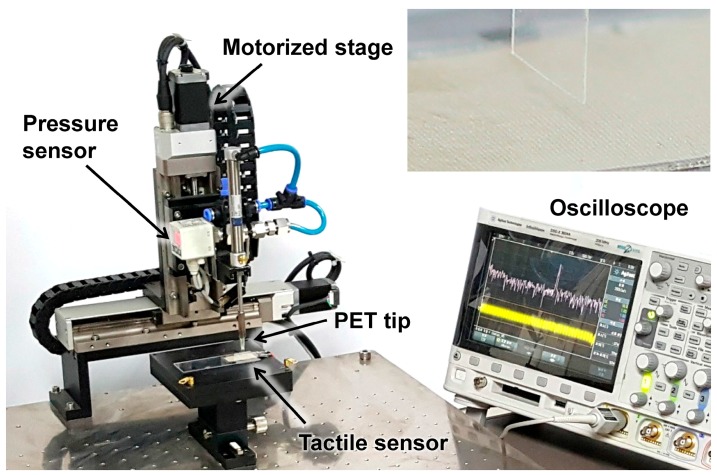
Optical image of the measurement set-up, where the inset shows an optical image of a SKIN and a hovering polyethylene terephthalate (PET) tip.

Figure 3a shows an SEM image of the fabricated SKIN #3. The fabricated SKIN was attached to a 28-μm-thick PVDF sensor (DT1-028K, Measurement Specialties, Hampton, VA, USA) to complete the flexible tactile sensor device structure (Figure 3b). The PVDF sensor was 12 mm wide and 30 mm long and had a piezoelectric voltage constant (g_33_) of −0.33 Vm/N. Figure 4 shows the measurement set-up, which had a 2-axis (x–z) motorized stage with 2 μm resolution. The scanning speed was controlled by the motorized *x* axis, and the contact depth was controlled by a motorized *z* axis. A PET tip with 5 mm of width and 125 μm in thickness was used for applying shear force to the ER (see inset). The PET tip was scanned across the SKIN surface without any vertical pressure, and the response voltage signal of the PVDF sensor was monitored using an oscilloscope. The tactile signal was obtained by subtracting the reference noise (which was obtained when the motorized stage was scanned above a sample without touching) from the measured PVDF signal in the frequency domain [29].

## 4. Tactile Sensitivity Dependent on SKIN Configuration

**Figure 5:**
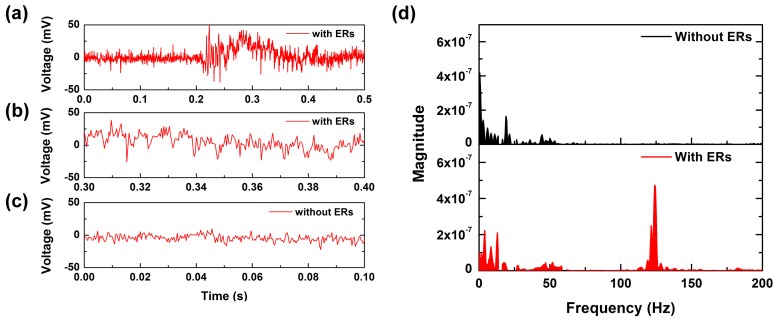
Measurement results of tactile sensor with or without epidermal fingerprint ridges (ERs) when a PET tip was scanned at 2.5 cm/s on the tactile sensor: (**a**) the polyvinylidene difluoride (PVDF) output signal of a tactile sensor with ridge structure; (**b**) the region of 0.3–0.4 s in (a); (**c**) the PVDF output signal of a tactile sensor without ridge structure; (**d**) the fast Fourier transform (FFT) results of (b,c).

The shear-induced torque applied to the ER structure acts to amplify the contact information [17,18]. To test the effect of having an ER on the sensor surface, the PET tip was scanned at 2.5 cm/s on the tactile sensor with SKIN #1 and the tactile sensor with only an SU-8 epidermal board without an ER. In the case of the tactile sensor with an ER, a significant fluctuation was observed in the sensor output signal after 0.2 s (see Figure 5a). Figure 5b shows the sensor signal between 0.3 and 0.4 s. It was observed that the main period of signal fluctuation was about 8 ms. However, in the case of the tactile sensor without ER, a periodic signal was not observed except for a 15-mV fluctuation induced by power noise (see Figure 5c). For more detailed analysis, we converted the time-dependent PVDF output signal to the frequency domain using fast Fourier transform (FFT). Figure 5d shows the denoised FFT results of Figure 5b,c. The tactile sensor without ERs showed frequency spectra in the low frequency range. This feature was induced by the stick-slip of the PET tip on the epidermal board. However, it was observed that there was a noticeable peak around 125 ± 5 Hz from the tactile sensor with an ER. Judging from the fact that *f* = *v*/*p*, where *f* is the signal frequency, *v* is the scanning speed, and *p* is the structural period, the distinct peak of about 125 Hz was induced by periodical interaction between the PET tip and the ER. This result shows that the ERs generate periodic contact information at a specific frequency. We defined this peak frequency induced by the period of ERs and the scanning speed as *f*_ER_.

**Figure 6:**
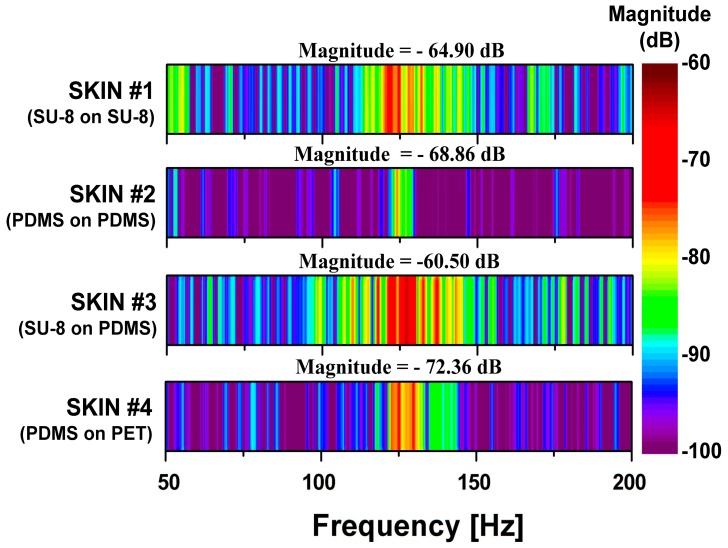
FFT results of tactile sensors with four types of SKINs when a PET tip was scanned at 2.5 cm/s on the tactile sensor. The indicated magnitude values show the magnitude of peak frequency (*f*_ER_) induced by a 200-μm period of ERs and a 2.5 cm/s scanning speed.

To test which SKIN layer composition had the most efficient transfer of surface interactions to the PVDF sensor, we performed the PET tip sliding measurement over the four different SKIN types and compared their FFT results. It can be seen in Figure 6 that all of the SKINs showed a peak in the FFT spectrum around *f*_ER_ = 125 Hz, which corresponded with the 2.5 cm/s scanning speed of the PET tip. The slight variation in the peak frequency may have been due to swelling in the polymer layers that was created during the different fabrication processes, causing expansion in the epidermal boards. In comparing the magnitudes of *f*_ER_, we found that SKIN #3 gave the highest magnitude of −60.50 dB, followed by SKIN #1 at −64.90 dB: both had SU-8 ERs. The harder SU-8 would have less elastic deformation than PDMS would, absorbing less of the lateral shear force applied by the sliding PET tip. Hence, as the PET tip slid over the SU-8 ERs, its interaction was more pronounced than with the SKINs with PDMS ridge structures, resulting in the signal magnitude of *f*_ER_ being 6.85 times higher for the sensor in SKIN #3 than in SKIN #2 (−68.86 dB), which both had PDMS epidermal boards. Comparing SKIN #3 and SKIN #1, the signal magnitude of *f*_ER_ was 2.75 times lower for the sensor with SKIN #1, which had the harder SU-8 epidermal board. This is understandable if one considers that a softer epidermal board would allow the ERs to tilt more with the applied shear force and create higher local deformation, while the harder SU-8 would distribute the vertical force over a wider area with lower local deformation, which would reduce the sensor signal. Therefore, it is reasonable that SKIN #4 (with soft PDMS ERs and a hard PET epidermal board) showed the lowest magnitude of *f*_ER_. The configuration of the material mechanical properties of SKIN #3 best mimicked that of the human epidermis (with harder fingerprint ridges on top of a softer epidermal board), which gave it the highest amplifying ability. From these results, we found that the human epidermal structure is designed not only to increase ductility but also to efficiently transfer the input stimulus on the skin to the mechanoreceptors.

**Figure 7:**
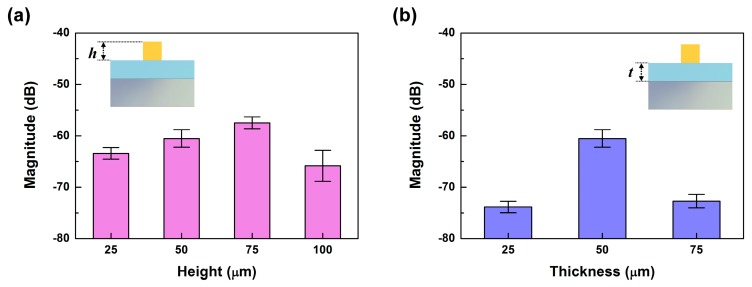
Dependence of the magnitude of *f*_ER_ on (**a**) the height (*h*) of the ER and (**b**) the thickness (*t*) of the epidermal board.

The heights of the ER structures could also affect the amount of shear force transferred to the PVDF sensor, since the generated torque would depend on the length of the displacement vector. To investigate the dependence of the sensor’s sensitivity on the height of the ER structure, we performed PET tip scanning measurements on the tactile sensor using SKIN #3, with ridges of 25 μm, 50 μm, 75 μm, and 100 μm in height. The *f*_ER_ of a pronounced peak appeared in the FFT results of all four tactile sensors at 125 Hz, corresponding to the scanning speed of 2.5 cm/s. Figure 7a shows the magnitude of *f*_ER_ of all four tactile sensors with different ER heights. The magnitude of *f*_ER_ increased by about 3.0 dB as the ER height increased from 25 μm to 75 μm, demonstrating that the increased height of the ER had an amplifying effect for shear sensing due to the increased torque. However, the magnitude of *f*_ER_ was greatly decreased at 100 μm in ER height. It seems that there was a limit to how high the ER could be made to take advantage of the amplifying effect, since beyond a critical ER height, or aspect ratio, we believe the ER structures may buckle and absorb the shear forces, leading to reduced force transfer. This height-dependent degradation has also been observed in other reports [30].

We also investigated the relationship between the tactile sensitivity and the thickness of the epidermal board. We used the same material configuration of SKIN #3 at a scanning speed of 2.5 cm/s, but with different board thicknesses varying between 25, 50, and 75 μm, and kept the height of the ER at 50 μm. We found that the highest magnitude of *f*_ER_ in the tactile sensor was from the 50-μm-thick epidermal board. This indicates that there may be an optimum thickness for the epidermal board that will give the highest force transfer while reducing the dispersion of the vertical force [31]. Due to these results, we chose SKIN #3 with an ER height of 75 μm and an epidermal board 50 μm thick for the following texture sensing.

## 5. Surface Period Detection

**Figure 8:**
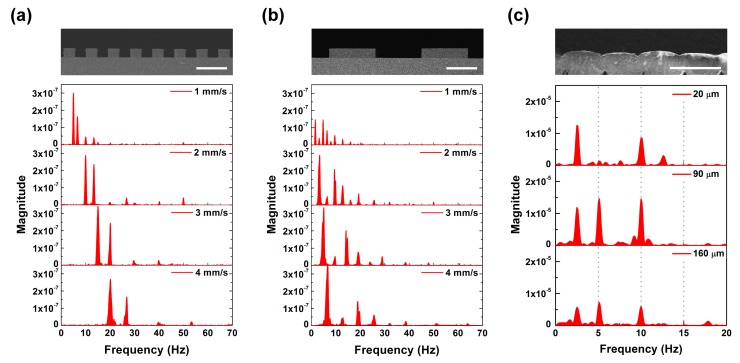
Surface period-detecting characteristics of biomimetic tactile sensor: the FFT results of the tactile sensor output induced by scanning the grating structures with (**a**) a 150-μm and (**b**) 625-μm period at 1–4 mm/s scanning speed, where the insets show cross-sectional SEM images of the grating structures (scale bars indicate 200 μm); (**c**) the FFT results of the tactile sensor output induced by scanning a 3D-printed structure at 2 mm/s (varying the contact depth), where the inset shows a cross-sectional SEM image of the 3D-printed structure (scale bar indicates 500 μm).

We evaluated the surface period-sensing characteristics of the developed tactile sensor using two periodic grating structures with a 150-μm and 625-μm period (insets of Figure 8a,b). The periodic grating structures were fabricated by a patterned 60-μm-thick SU-8. Figure 8a,b shows the FFT results of tactile sensor output induced by scanning performed at 1–4 mm/s. When the grating structure slid on the tactile sensor, individual ERs periodically interacted with each grating structure, and simultaneously the individual grating structure periodically interacted with each ER. Therefore, the FFT results present the *f*_ER_ and the peak induced by a surface period of a contact object. We observed that the distribution of the peaks showed a blue shift as the scanning speed increased, with the scanning speed divided by the highest peak frequency remaining the same. At a 2-mm/s scanning speed (Figure 3a), a distinct peak for *f*_ER_ was observed at 10 Hz, and peaks at 13.3 Hz were induced by the 150-μm period of the surface grating structure, with harmonic signals appearing at each 10- and 13.3-Hz multiple. We saw similar characteristics for the 625-μm periodic structure. This showed that our sensor was able to detect surface structures under 100 μm (75 μm wide and 60 μm high). Since the highest peak would come from the ER’s interaction with the contacting surface, the *f*_ER_ offered information on scanning speed. Then the other peaks in the spectra could elucidate the surface periods of the object with the known scanning speed.

In sensing the surface roughness, varying the contact depth was an important factor in measuring the surface structure with different heights. A plastic (polylactic acid) surface with a 390-μm-period elliptic structure with a 20-μm height difference was fabricated by using a fused filament fabrication-type 3D printer, as seen in the inset of Figure 8c. The reciprocating direction of the printing nozzle resulted in the height difference. Figure 8c shows the FFT results of the tactile sensor when the printing structure was scanned at 2 mm/s, increasing the contact depth. Comparing the FFT result at a 90-μm contact depth to that at 20 μm, we observed an additional peak at 5.12 Hz, reflecting the period of the printing structure with a lower height. The peak at 5.12 Hz was not considered to be a harmonic of 2.56 Hz due to its higher magnitude. This result shows that our tactile sensor was able to detect not only the surface period but also a 20-μm height difference in surface structure. In the deeper contact depth of 160 μm, the signal magnitude of FFT decreased. It seems that the ER was not able to deform and recover fast enough due to the increased pressure resulting from the increased contact depth.

**Figure 9:**
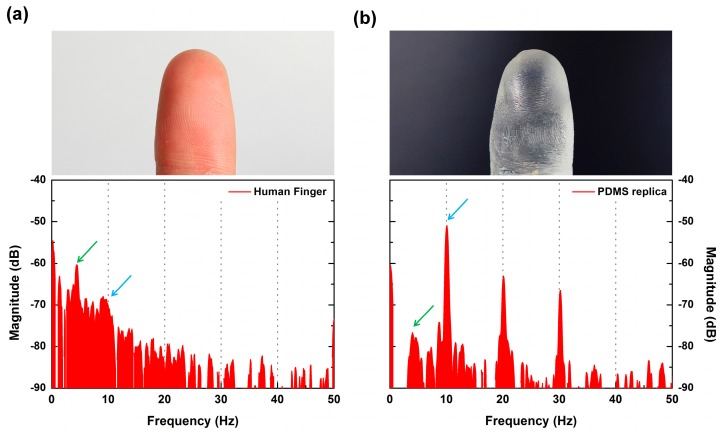
FFT results of tactile sensor output induced by scanning (**a**) a human finger and (**b**) a PDMS replica, where the insets show optical images of a human finger pad and the PDMS replica. Blue arrows indicate the *f*_ER_, and green arrows indicate the peaks of ERs of the contact objects.

When the SKIN interacts with the object’s surface, the generated vibration should depend on the frictional aspect of the materials involved. Therefore, even with the same surface roughness, if the material is different, it should produce vibrations with different spectral distributions. We fabricated a PDMS replica of a finger, which should have had identical surface structures to the actual finger used, and measured the sensor signal through tactile scanning to see how the measured signals would differ. Figure 9a,b shows the FFT results, and it is clear that the two results were quite different and distinguishable. In both cases, distinct peaks were observed identically at *f*_ER_ = 10 Hz (representing the ER period) and at 4.2 Hz (which was dependent on the 480-μm period of the human fingerprint). However, differences in the amplitudes of major peaks and their harmonics and spectral distribution in the lower frequency region were observed. This difference seems to have been caused by the stiffness difference between the human finger and the PDMS replica. The human finger showed larger deformability due to its Young’s modulus (~100 kPa [25]) being lower than PDMS (750 kPa). The human skin’s lower modulus led to a larger contact area and higher friction with the SKIN layer of the sensor. This allowed the characteristic peaks to become distributed, forming a higher amplitude spectrum in the low-frequency region. In addition, the chemical characteristics of a finger surface (cell membrane, oil, and sweat) differ from those of PDMS, which produces differing frictional characteristics. This result showed that our sensor was able to distinguish between different materials even if they had the same surface structure.

## 6. Texture Detection

**Figure 10:**
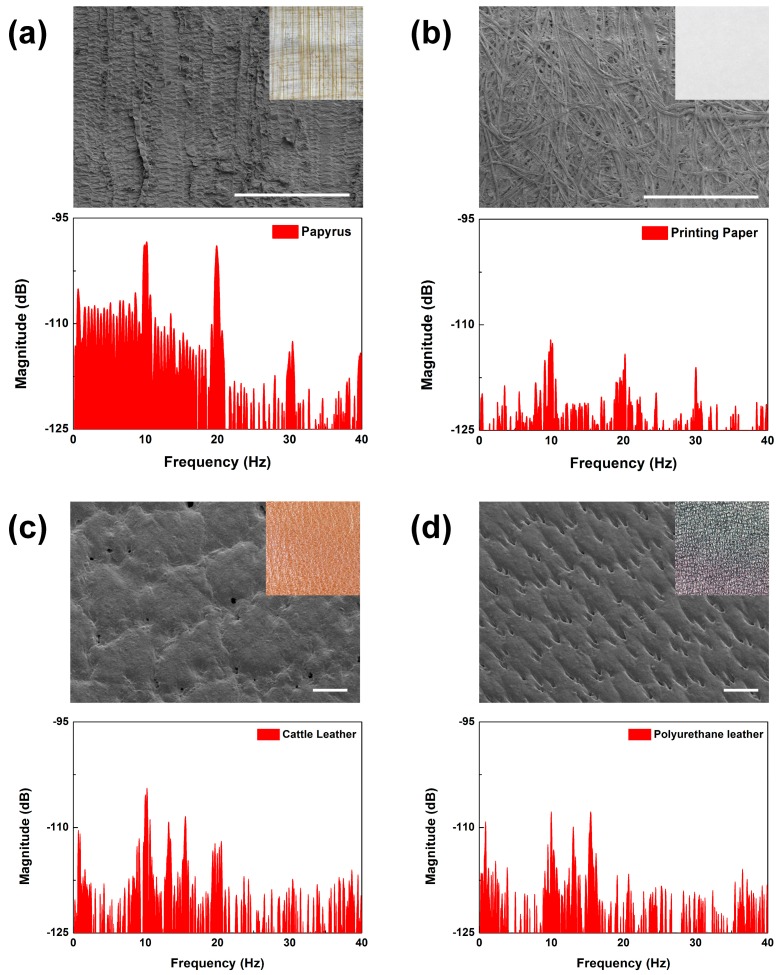
FFT results of tactile sensor output induced by scanning (at a 2-mm/s scanning speed) (**a**) a sheet of papyrus, (**b**) a sheet of printing paper, (**c**) cattle leather, and (**d**) polyurethane artificial leather, where insets show SEM and optical images of each contact object (scale bars indicate 500 μm).

The ability to discern surface feature periods and contact materials may be used to distinguish the characteristics of similar materials. We performed surface scanning measurements on similar materials with different compositions to test this ability of our biomimetic tactile sensor. First, we compared the scanning results between a sheet of printing paper and a sheet of papyrus at a 2 mm/s scanning speed (see Figure 10a,b). The results taken from the printing paper scanning showed that the peaks at *f*_ER_ and its integral multiples showed far lower amplitudes than did papyrus. These complex spectra could be simply analyzed using the energy spectrum density (ESD), which is the integral of FFT in a specific frequency range. Comparing the two results in the frequency range under and over *f*_ER_, the ESDs of the papyrus were 22 times and 13 times larger than those of the printing paper. It can be said that the papyrus had more surface features with periods shorter and longer than 200 μm. This can be verified visually (as shown in the optical and SEM images of Figure 10a,b), as the printing paper was featureless while the papyrus was feature-rich with many observable line structures. The surface features of the papyrus had higher directionality, periodicity, and height than did the printing paper. Due to these structural differences in surface topology, papyrus had the higher peak amplitude over most of the observed frequency range. Since our fingertips felt that the papyrus surface was “rougher” than that of the printing paper, the higher peaks in the FFT results may reflect the roughness of the material surface.

For real leather and artificial leather, it is quite difficult for an untrained person to distinguish between the two just by scanning their fingers over the surfaces. We found that the scanning results of these materials did produce FFT results that were discernable. As shown in the inset images of Figure 10c,d, cattle leather and polyurethane leather have similar surface structures with many grains and pores. The FFT results showed a similar spectral distribution, which made it understandable why it would be difficult to distinguish between them just by relying on touch. However, there were features that stood out. In the frequency range from 8 to 22 Hz, the ESD of the cattle leather was 2 times larger than that of the polyurethane leather. This shows that our sensor may be used to detect fine differences between artificial and real leather.

**Figure 11:**
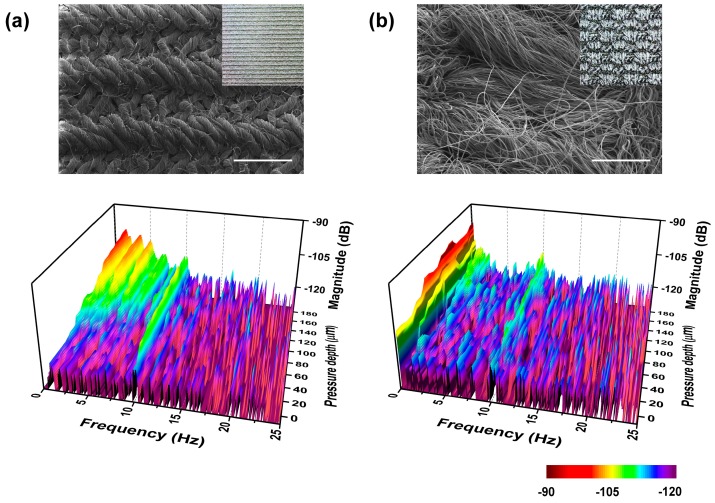
FFT results of tactile sensor output induced by scanning (**a**) a hard fabric with tight weaves and (**b**) a soft fabric with hair-like structures, increasing the contact depth. Inset images show SEM and optical images of the two different fabrics (scale bars indicate 1 mm).

The results of Figure 8c show that the contact depth was an essential factor in detecting more detailed surface characteristics. Varying the contact depth, we measured the change in the frequency spectrum between two fabrics with different hardness. As seen in the optical image of Figure 11a,b, the harder fabric had tight weaves and the softer fabric had hair-like structures. The topmost spectrum was recorded when the 10-Hz fingerprint peaks started to appear. Then the fabrics were scanned with the sensor pressed on its surface with the pressure depth increasing in 20-μm steps toward a 200-μm total depth. In the low-contact depth, we observed similar frequency spectra, which showed no specific peak except for *f*_ER_ despite the significant visual differences between the two fabrics. Increasing the contact depth, we observed the appearance of peaks at 1.6 Hz and 0.2 Hz and their harmonics with a 1.2-mm period of the hard fabric and a 10-mm period of the soft fabric. Comparing the two FFT results, the harder-fabric FFT showed more distinct peaks appearing as the scanning pressure increased to that of the softer-fabric FFT. We can conclude that the frequency spectra taken at various contact depths can give additional hardness information, which will aid in increasing the ability of the biomimetic tactile sensor to distinguish between various materials and which may make tactile sensor-based material identification a reality.

## 7. Discussion

Previously reported tactile sensors that were based on measuring scanning vibrations commonly used accelerometers and microphones [32,33]. These sensors were built into or on top of an artificial finger, and vibration damping could occur, which would reduce the accuracy of the results. Since our biomimetic tactile sensor would be in direct contact with an object, it could effectively detect the vibration information induced by the interaction between the ERs and the contact object without damping. Our SKIN showed tactile sensitivity that was high enough to detect sub-100-μm surface structures of a sheet of papyrus. It has been reported that ERs show good tactile sensitivity on surfaces with structural periods that are 0.5–2 times as long as that of the ERs [26]. When varying the design of the ER period, the tactile sensitivity may be adjusted to the target sensitivity, which may be required for various applications.

Conventional fingerprint identification security systems can detect the surface structure of human finger pads but cannot discriminate between finger pad replicas. In the results in Figure 9, it is shown that our biomimetic tactile sensor had the capability of discriminating between a human finger pad and its replica, which may make them applicable for future security systems. By utilizing its mechanical flexibility, if we incorporate the biomimetic tactile sensor into the previously reported tactile stiffness sensor [34], it may be possible to develop a portable tactile measurement system that can detect surface roughness as well as stiffness, which will make tactile surface recognition possible.

## 8. Conclusions

We developed a SKIN with a bilayer structure with different elastic moduli that mimics human ER and epidermis characteristics. When a hard ER and a soft epidermal board were used for the SKIN, we improved the effectiveness of transferring surface shear forces into vertical vibrations. The SKIN with a hard SU-8 ER and a soft PDMS epidermal board showed 6.85 times higher tactile sensitivity than did the one fabricated using only the soft PDMS. We optimized the ER dimensions and the epidermal board thickness, and the biomimetic tactile sensor with the optimized SKIN was able to detect a 75-μm period and a 20-μm height difference in a contact surface. Our sensor also showed an ability to sense the difference between contact with a human finger and a PDMS replica with the same surface structure. In addition, we demonstrated that it was possible to discern the differences in textures of paper, leather, and fabric using the biomimetic tactile sensor. With further development and through use of the sensor’s mechanical flexibility, our biomimetic tactile sensor may make texture-recognizing artificial fingers a possibility.
